# Mild chemistry synthesis of ultrathin Bi_2_O_2_S nanosheets exhibiting 2D-ferroelectricity at room temperature†

**DOI:** 10.1039/d4sc00067f

**Published:** 2024-04-09

**Authors:** Riddhimoy Pathak, Prabir Dutta, Kapildeb Dolui, Aastha Vasdev, Adrija Ghosh, Raj Sekhar Roy, Ujjal K. Gautam, Tapas Kumar Maji, Goutam Sheet, Kanishka Biswas

**Affiliations:** a New Chemistry Unit, International Centre for Materials Science, School of Advanced Materials, Jawaharlal Nehru Centre for Advanced Scientific Research (JNCASR) Jakkur P.O. Bangalore 560064 India kanishka@jncasr.ac.in; b Department of Materials Science & Metallurgy, University of Cambridge 27 Charles Babbage Road Cambridge CB3 0FS UK; c Department of Physical Sciences, Indian Institute of Science Education and Research Mohali Sector 81, S. A. S. Nagar, Manauli, P.O. Box 140306 India; d Department of Chemical Sciences, Indian Institute of Science Education and Research Mohali Sector 81, S. A. S. Nagar, Manauli, P.O. Box 140306 India; e Chemistry and Physics of Materials Unit, Jawaharlal Nehru Centre for Advanced Scientific Research (JNCASR) Jakkur P.O. Bangalore 560064 India

## Abstract

Modern technology demands miniaturization of electronic components to build small, light, and portable devices. Hence, discovery and synthesis of new non-toxic, low cost, ultra-thin ferroelectric materials having potential applications in various electronic and optoelectronic devices are of paramount importance. However, achieving room-temperature ferroelectricity in two dimensional (2D) ultra-thin systems remains a major challenge as conventional three-dimensional ferroelectric materials lose their ferroelectricity when the thickness is brought down below a critical value owing to the depolarization field. Herein, we report room-temperature ferroelectricity in ultra-thin single-crystalline 2D nanosheets of Bi_2_O_2_S synthesized by a simple, rapid, and scalable solution-based soft chemistry method. The ferroelectric ground state of Bi_2_O_2_S nanosheets is confirmed by temperature-dependent dielectric measurements as well as piezoelectric force microscopy and spectroscopy. High resolution transmission electron microscopy analysis and density functional theory-based calculations suggest that the ferroelectricity in Bi_2_O_2_S nanosheets arises due to the local distortion of Bi_2_O_2_ layers, which destroys the local inversion symmetry of Bi_2_O_2_S.

## Abstract 2

Synthesis of room temperature ferroelectric 2D Bi_2_O_2_S ultra-thin nanosheets through a mild chemistry route.

## Graphical abstract

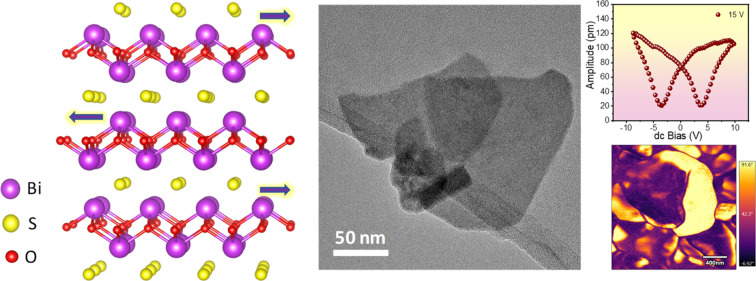

## Introduction

Since the discovery of graphene in 2004,^1^ scientists are driven to find novel atomically thin two dimensional (2D) layered materials showing striking optical,^2–5^ electrical,^6–8^ thermoelectric,^9,10^ and mechanical^11^ properties. The intriguing aspect of these properties measured in 2D materials is that they differ significantly from their bulk counterpart. However, experimental realization of room-temperature ferroelectricity in ultra-thin 2D materials is extremely challenging but it is of paramount importance for electronics and optoelectronics like random access memory, logic devices,^12–14^*etc.*

Conventional three-dimensional ferroelectric materials like BaTiO_3_ and PbTiO_3_ lose their ferroelectricity when the thickness is brought down below a critical value owing to the depolarization field.^15–17^ Nevertheless, there are several theoretical predictions of ferroelectricity in 2D materials such as monolayers of molybdenum and tungsten dichalcogenide families (MoX_2_ and WX_2_ where X = S/Se/Te), a few layers of phosphorene,^18^ SnSe,^18^ SnTe,^19^ GeSe,^20^ PbTe,^21^ In_2_Se_3_,^22^ CuInP_2_S_6_,^23^ bismuth oxychalcogenides,^24^*etc.* Many of the mentioned materials have a centrosymmetric space group in their bulk state; however, when they are brought down to below a critical thickness, the local symmetry breaking may give rise to ferroelectricity. Till date, among the several theoretically predicted 2D ferroelectric materials, SnTe,^25^ WTe_2_,^26^ SnS,^27^ CuInP_2_S_6_, 1T-MoTe_2_,^28^ α and β In_2_Se_3_ ^29,30^ and Bi_2_O_2_Se^31^ with a few nanometer thickness have been experimentally verified to show ferroelectricity.

**Fig. 1:**
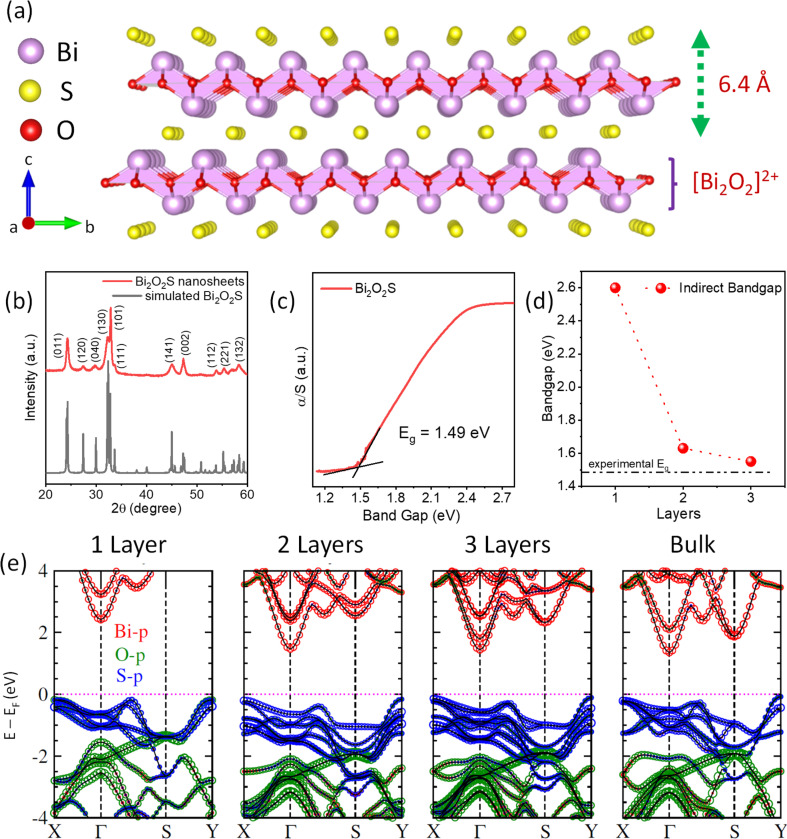
(a) Crystal structure of Bi_2_O_2_S showing the charged heterostructure layers. (b) Room temperature X-ray diffraction (XRD) pattern of the synthesized Bi_2_O_2_S nanosheets. (c) Room temperature band gap data of the synthesized Bi_2_O_2_S powders. (d) Variation of the indirect band gap with layers of Bi_2_O_2_S as per DFT calculations. (e) Electronic band structure of mono-, bi-, tri-layer and bulk Bi_2_O_2_S.

Bismuth oxychalcogenides are zipper-like 2D materials and can be employed as a substitute for mainstream van der Waal (vdW) 2D materials due to their superior electronic and optoelectronic properties.^32^ Among the different members of the oxychalcogenide family, 2D Bi_2_O_2_Se showed tremendous promise in opto-electronics.^33–35^ The carrier mobility of 2D Bi_2_O_2_Se rises to a gigantic value of >20 000 cm^2^ V^−1^ s^−1^ at 2 K and thus can be used as a field-effect transistor and other logic devices at cryogenic temperatures.^36^ The high carrier mobility further results in superior photoresponsivity in 2D Bi_2_O_2_Se.^37^ Similarly, high electrical conductivity is seen in Bi_2_O_2_Se along with low thermal conductivity because of its layered structure, making Bi_2_O_2_Se a brilliant candidate for thermoelectric applications.^38,39^ Recently, Bi_2_O_2_Se nanosheets are reported to show ferroelectricity due to the orthorhombic distortion of the Bi_2_O_2_ layer.^31^ However, the major drawback of Bi_2_O_2_Se for the mass-market application is its toxicity due the presence of Se as well the low abundance of Se in the world.^40^ The sulphur analogue, Bi_2_O_2_S, belongs to the same family, which is an environment-friendly and lower cost substitute of selenium analogues. While the crystallographic *a* and *b* axes of Bi_2_O_2_Se are equal in length making it a tetragonal structure (*I*4/*mmm*), Bi_2_O_2_S has a slight difference in its *a* and *b* axes, and thus, it crystallizes in an orthorhombic structure (*Pnnm*).^24^ Although there are only a few reports on near-infrared photodetectors^41,42^ and photocatalytic CO_2_ reduction^43^ based on Bi_2_O_2_S nanostructures, the ferroelectric or multiferroic properties of 2D ultra-thin Bi_2_O_2_S have not been yet explored experimentally. Bismuth oxysulphide (Bi_2_O_2_S) exhibits a charged layer heterostructure with [Bi_2_O_2_]^2+^ and S^2−^ stacked together with a gap of 6.4 Å (Fig. 1a). However, the interlayer displacement of two corresponding Bi_2_O_2_ layers results in a minute anisotropy in the *x* and *y* directions. This results in a change in the crystal structure of Bi_2_O_2_S to orthorhombic with the space group *Pnnm* (*a* = 3.85 Å, *b* = 3.89 Å and *c* = 11.97 Å).

Herein, we have synthesized ultrathin freestanding nanosheets of Bi_2_O_2_S *via* a simple, rapid, and scalable solution-based mild chemistry method at room temperature. Atomic force microscopy (AFM) studies confirm that the thickness of the sheet is nearly 2 nm, which corresponds to three layers of Bi_2_O_2_S. Transmission electron microscope (TEM) images confirm that the lateral dimensions of the nanosheets vary from 100 to 200 nm. Temperature-dependent dielectric measurement and differential scanning calorimetry (DSC) studies indicate a ferroelectric like transition at 440 K in the case of Bi_2_O_2_S nanosheets. Piezoelectric force microscopy (PFM) performed at room temperature indicates the presence of local ferroelectric domains in Bi_2_O_2_S nanosheets. PFM Dual ac Resonance Tracking Spectroscopy (PFM DART) indicates a clear signature of spontaneous polarization and its 180° switching behavior under the application of an external electric field confirms the ferroelectric nature of Bi_2_O_2_S nanosheets. To understand the underlying cause of ferroelectricity in the Bi_2_O_2_S nanosheets, we performed high resolution TEM (HRTEM) analysis and supported our findings with density functional theoretical (DFT) calculations of phonon dispersion. The observed spontaneous polarization in Bi_2_O_2_S nanosheets originates from the local distortion of Bi_2_O_2_ layers, which breaks the inversion symmetry locally. Our DFT calculations verify that each layer of Bi_2_O_2_ is ferroelectric in nature due to this spontaneous distortion. However, two consecutive layers have an antiferroelectric interaction among them owing to the polarization in the opposite direction. Thus, dipole moments are spontaneously generated for an odd number of layers in Bi_2_O_2_S, which give rise to lingering ferroelectricity in Bi_2_O_2_S nanosheets.

## Results and discussions

A mild chemistry or soft chemistry or chimie douce approach has been previously utilized to synthesize many exotic non-thermodynamic phases of metal oxides and sulfides,^44–46^ and even recently to synthesize high entropy perovskite halide single crystals.^47^ Here, ultra-thin single-crystalline nanosheets of Bi_2_O_2_S were synthesized by a facile and rapid solution-based mild chemistry reaction of bismuth nitrate pentahydrate (Bi(NO_3_)_3_·5H_2_O) and thiourea (CS(NH_2_)_2_) (details in the ESI†) at room temperature. The nanosheets are free-standing and do not require a substrate to grow on. The total reaction time is very fast, *i.e.*, ∼10–15 min and the reaction can be performed in a standard laboratory beaker at room temperature. In the first step, Bi(NO_3_)_3_·5H_2_O gets hydrolyzed in the presence of an aqueous medium to form milky white BiONO_3_ and the process accelerates after the addition of KOH. CS(NH_2_)_2_ was used as a precursor for sulphide ions since it can slowly release S^2−^ ions in the basic medium. With the addition of CS(NH_2_)_2_ in the basic medium, it decomposes into sulphide ions (S^2−^) that react with BiONO_3_ to form an orange-brown coloured Bi_2_O_2_S precipitate. The reaction is scalable up to ∼1 g under ambient laboratory conditions. Thus, Bi_2_O_2_S is synthesized by bringing together [Bi_2_O_2_]^2+^ and S^2−^ from two different precursors in an alkaline medium and they electrostatically combine to form Bi_2_O_2_S. We have synthesised a controlled bulk phase of Bi_2_O_2_S by a vacuum sealed-tube solid state melting reaction at 1223 K by mixing stoichiometric amounts of Bi_2_O_3_ and Bi_2_S_3_ (see the Methods in the ESI†).

The room temperature powder X-ray diffraction (PXRD) pattern of the as-synthesized Bi_2_O_2_S nanosheets before vacuum drying shows a diffuse broad pattern with major peaks at 2*θ* ∼ 27° and 40° (Fig. S1, ESI†). The broadness and diffuse nature of the XRD peaks give us an impression of the nanocrystalline nature of the synthesized sample. After subjecting the powder to a 36 hour vacuum drying process, the peaks in the XRD pattern were easily identifiable and could be effectively matched with a simulated pattern of polycrystalline Bi_2_O_2_S (Fig. 1b). This indicates pure phase synthesis of Bi_2_O_2_S nanosheets with an orthorhombic structure (space group *Pnnm*). However, even after vacuum drying the peaks were substantially broad, and majority of the peaks were overlapped indicating that the nano-dimension morphology has been retained after vacuum drying. The room temperature band gap of Bi_2_O_2_S was measured to be ∼1.5 eV (Fig. 1c). We compared the experimentally obtained band gap with the DFT calculated band gap of mono-, bi- and tri-layer Bi_2_O_2_S (Fig. 1d). The valence bands are composed of p orbitals of O and S, where the latter occupy the upper position due to the difference in electronegativity and the conduction band is mainly contributed by the p orbitals of Bi (Fig. 1e). The DFT calculations also suggest that there is an enhancement of the band gap at the *Γ* point upon a decrease in the number of layers. This arises due to the quantum confinement and the bandgap reaches a value of 2.6 eV for monolayer Bi_2_O_2_S. With the increase in the number of layers in Bi_2_O_2_S, there is an emergence of higher energy bands along the *Γ*–*S* direction as well as the *S* point, resulting in the formation of an indirect band gap along this region of the Brillouin zone. Additionally, the experimental bandgap closely matches with that of trilayer Bi_2_O_2_S obtained through DFT calculations. This result hints at the possibility of the existence of nanosheets, potentially consisting of just a few layers.

**Fig. 2:**
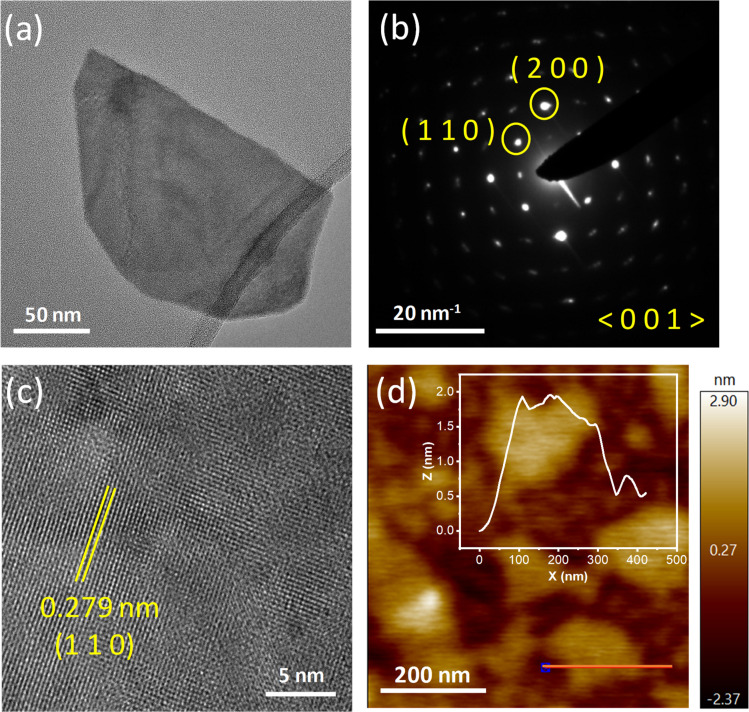
(a) Transmission electron microscopy (TEM) image of a single Bi_2_O_2_S nanosheet. (b) Selected area electron diffraction (SAED) pattern of the nanosheet along the 〈001〉 zone axis. (c) High resolution transmission electron microscopy (HRTEM) image of the nanosheet showing the (110) plane. (d) Atomic force microscopy (AFM) image of the Bi_2_O_2_S nanosheets.

To get an idea about the morphology of the synthesized Bi_2_O_2_S sample, transmission electron microscopy (TEM) and high-resolution transmission electron microscopy (HRTEM) were performed. Fig. 2a, S2a and S3a, ESI,† show the presence of Bi_2_O_2_S nanosheets. The selected area diffraction pattern (SAED) indicates the single-crystalline nature of the sheets (Fig. 2b and S2b, ESI†). The lateral dimension of the nanosheets was approximately in the range of 150–300 nm. Fig. 2c shows the HRTEM image of single crystalline Bi_2_O_2_S. The *d* spacing obtained in the HRTEM image is around 0.279 nm, which corresponds to the (110) plane of Bi_2_O_2_S. The nanosheets of Bi_2_O_2_S are very thin and the AFM images substantiate this claim (Fig. 2d, S4a and S5†). The thickness of the sheets is around 1.9–2 nm, which corresponds to approximately three layers of Bi_2_O_2_S confirming the thin nanosheet morphology of the synthesized sample which is further corroborated through the amplitude image (Fig. S4b, ESI†). The presence of Bi, O and S is corroborated through energy dispersive X-ray (EDX) color mapping (Fig. S3b–d†). X-ray photoelectron spectroscopy (XPS) on Bi_2_O_2_S (Fig. S6†) confirms the +3 and −2 oxidation states of Bi and S. The characteristic 1s peak of O at 532 eV was observed along with the 4f_7/2_ and 4f_5/2_ characteristic peaks of Bi at 159 eV and 164 eV, which match with the previously reported^48,49^ values of O^2−^ and Bi^3+^. The characteristic peaks of S^2−^, *i.e.* 2p_3/2_ and 2p_1/2_, are found at 160 and 161 eV.

To gain insight into the bonding nature of the Bi_2_O_2_S nanosheets, Raman spectroscopy was conducted at room temperature and subsequently compared with its bulk counterpart (Fig. S7†). There are a few discrepancies regarding the position of A_g_ mode in Bi_2_O_2_S. Some research groups state that it is present above 210 cm^−1^, whereas others claimed that it is seen below 200 cm^−1^.^50,51^ Our results are similar to the findings of Xu *et al.* considering harmonic approximation.^41,42,50^ On further detailed analysis, it is seen that the B_3g_, B_1g_ and A_g_ Raman peaks of Bi_2_O_2_S nanosheets (66 cm^−1^, 92 cm^−1^ and 182 cm^−1^, respectively) are slightly low energy shifted compared to the Raman peaks of bulk Bi_2_O_2_S. The lower energy shift in the Raman peaks can be attributed to phonon softening, resulting either from weakened interlayer coupling or an increase in strain when Bi_2_O_2_S is reduced to nano-dimensions.^52,53^

**Fig. 3:**
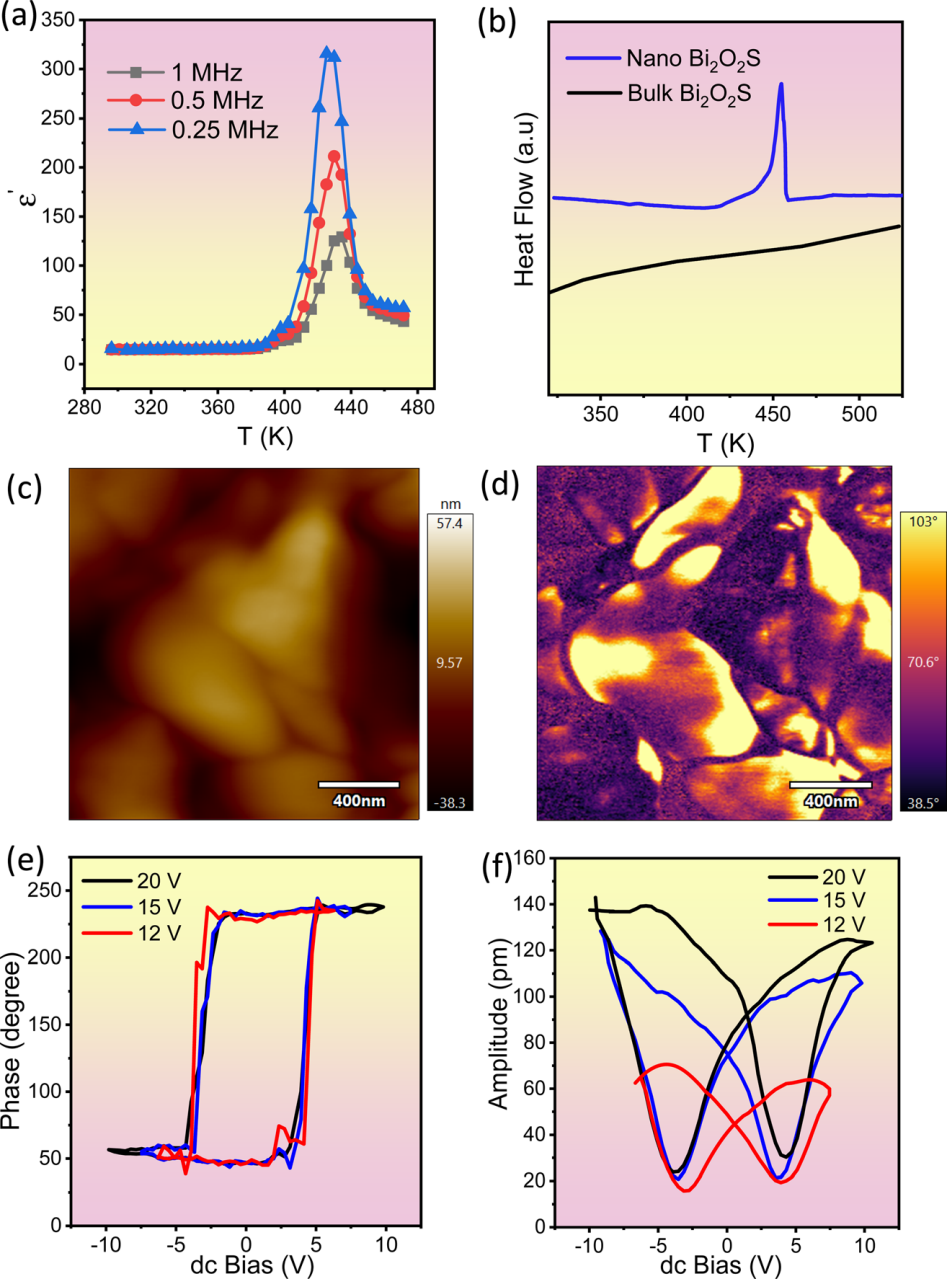
(a) Temperature variation of the real part of the dielectric constant (*ε*′) of Bi_2_O_2_S nanosheets at 1 MHz, 0.5 MHz and 0.25 MHz. (b) Comparative differential scanning calorimetry (DSC) data of bulk and nanosheets of Bi_2_O_2_S. (c) Topography image of drop casted Bi_2_O_2_S nanosheets. (d) Phase images of Bi_2_O_2_S nanosheets obtained through piezoelectric force microscopy (PFM). (e) Phase signal and (f) amplitude signal of Bi_2_O_2_S nanosheets obtained through switching spectroscopy PFM.

We have measured the temperature dependent dielectric properties of both nanosheet and bulk Bi_2_O_2_S. The real part of relative permittivity (*ε*′) shows a peak near 430 K without any significant dependence on the applied frequency (Fig. 3a), which can be attributed to a paraelectric to ferroelectric like transition. The maximum values of *ε*′ are 315, 212 and 129 at 0.25, 0.5 and 1 MHz, respectively. Further, in differential scanning calorimetry (DSC) of the nanosheet sample the peak at around *T*_c_, ∼440 K, substantiates this claim (Fig. 3b). Interestingly, no such peak is seen in bulk Bi_2_O_2_S, suggesting that the ferroelectric ground state is stabilized when the material is synthesised in the form of 2D ultra-thin nanosheets.

To further confirm the ferroelectric instability in the 2D nanosheets, switching spectroscopy by piezoelectric force microscopy (PFM) was performed at room temperature on nanosheets of Bi_2_O_2_S. The topographic (Fig. 3c and S8a, ESI†), phase (Fig. 3d and S8b, ESI†) and amplitude images (Fig. S8c and S9, ESI†) of the Bi_2_O_2_S nanosheets indicate the presence of local ferroelectric domains within it with invariable electrical polarization. The topographic image indicates the topography or morphology of Bi_2_O_2_S nanosheets spin-coated on an indium tin oxide (ITO) substrate. The topography and phase images obtained from PFM analysis are the evidence for the existence of local ferroelectric domains with uniform electrical polarization. The occurrence in the contrast difference in the phase image denotes the occurrence of oppositely polarized local neighboring ferroelectric domains. The corresponding amplitude image (Fig. S9, ESI†) demonstrates the amplitude of the ferroelectric domains as depicted in the phase image. To further establish the presence of local ferroelectric domains in Bi_2_O_2_S nanosheets, spontaneous polarization state and switching behavior under an externally applied field were studied and the observed room temperature hysteric behavior in the “off-state” piezoelectric signal of Bi_2_O_2_S nanosheets is shown in Fig. 3e and f. The ‘off-state’ amplitude suggests the enhancement of the displacement amplitude, *i.e.* the electromechanical response of the sample with increase in the applied voltage. The phase of the PFM responsive signal (Fig. 3e) indicates the direction of the polarization of the nanodomains, while the amplitude of the PFM signal (Fig. 3f) indicates the magnitude of the polarization of the nanodomains. The polarization hysteresis depicted in the PFM DART phase data (Fig. 3e), which arises because of 180° switching, is the fingerprint of ferroelectric materials and involves the nonlinear increase of polarization with the electric field. The coercive field of the measured Bi_2_O_2_S nanosheets is 8 V, indicating that the energy barrier between opposite polarized states is moderately high. However, the coercive field found in Bi_2_O_2_Se nanosheets reported by Ghosh *et al.* is found to be 14 V (ref. 31) indicating that the ferroelectric nature in Bi_2_O_2_S is relatively weaker than that of Bi_2_O_2_Se. Additionally, this type of reorientation or switching behaviour is well known to contribute to the strain of these materials. As a result, if the electric field is cycled, a strain-electric field hysteresis loop is obtained, which resembles the shape of a butterfly as presented in the PFM DART amplitude data (Fig. 3f). The butterfly loop observed in the amplitude *vs.* dc bias plot indicates the ability to switch between two stable polarizable states under the influence of an external bias field, confirming the ferroelectric nature of Bi_2_O_2_S 2D nanosheets.

**Fig. 4:**
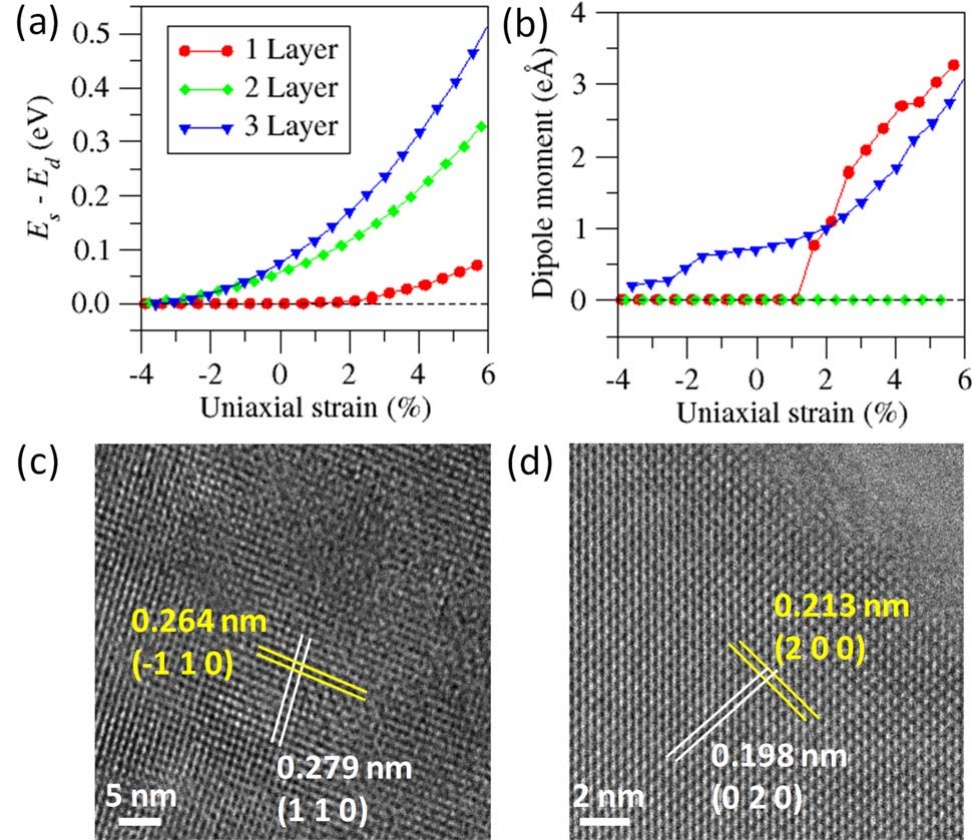
(a) Variation of *E*_s_ − *E*_d_ and (b) total dipole moment under strain for monolayer, bilayer, and trilayer Bi_2_O_2_S nanosheets (*E*_s_ and *E*_d_ represent the total energy for symmetrical and distorted structures, respectively). (c) and (d) HRTEM image projected along the [001] direction exhibiting deviation between interatomic distances along (110) & (−110) and (200) & (020) directions.

As mentioned earlier, bulk Bi_2_O_2_S crystallizes in a centrosymmetric space group *Pnnm*, so the presence of ferroelectric domains in Bi_2_O_2_S 2D nanosheets is rather surprising. Therefore, to further comprehend the origin of the observed ferroelectricity in the 2D nanosheets, density functional theory (DFT) calculations were performed for a symmetric structure (*E*_s_) as well as distorted structure (*E*_d_), in which Bi and O/S atoms were displaced diagonally from their equilibrium positions in opposite directions. We have carried out further calculation in the Bi_2_O_2_S system by applying compressive uniaxial strain along the crystallographic *a* direction. The extent of the strain has been measured as *ζ* = (*a* − *a*_0_)/*a*_0_, where a and *a*_0_ are the in-plane lattice constants for the strained and pristine nanosheets, respectively. Fig. 4a and b show the variation of *E*_s_ − *E*_d_ and the dipole moment with the applied uniaxial strain along the *a* axis for mono- to tri-layer Bi_2_O_2_S. Our calculated data show that the ferroelectricity in Bi_2_O_2_S exists for *ζ* > 2%, which is slightly higher than that of Bi_2_O_2_Se.^31^ Bulk Bi_2_O_2_S has a (BiO)_2_ rhombic network (*D*_2h_), which creates the [Bi_2_O_2_]^2+^ layer. When the dimension of Bi_2_O_2_S is diminished to the nano-regime, the strain results in the atomic displacement of Bi and O, which destroys the rhombic network locally. The destruction of the rhombic structure breaks the inversion symmetry locally in the Bi_2_O_2_S nanosheet.

We find that at different *ζ*, the values of *E*_s_ − *E*_d_ are different for mono-, bi- and tri-layer Bi_2_O_2_S (Fig. 4a). Additionally, Δ*E* (=*E*_s_ − *E*_d_) becomes positive upon application of a minute strain (2%), indicating that the spontaneous distortion of the Bi and O atoms is energetically favourable. Interestingly, for trilayer Bi_2_O_2_S, we get a spontaneous dipole moment even in the unstrained phase, which is not seen for monolayers and bilayers indicating ferroelectric behaviour (Fig. 4b). In the case of a single layer of Bi_2_O_2_S, a small amount of strain of around 1.6% is required to stimulate ferroelectricity. For bilayers of Bi_2_O_2_S, surprisingly, the dipole moment is zero for all the calculated strain values even though there is a finite Δ*E* value for the bilayer. We propose that the residual dipole moment that is generated in a single layer of Bi_2_O_2_S due to the spontaneous distortion of Bi and O couples with the following layer of residual dipole moment, in an antiferroelectric manner thus producing no net dipole moment in the bilayer. Thus, an even number of layers of Bi_2_O_2_S will show no net dipole moment and an odd number of layers will show a considerable amount of ferroelectric behaviour due to the lingering dipole moment.

To verify the spontaneous local distortion experimentally, we have carried out high-resolution transmission electron microscopy (HRTEM) of Bi_2_O_2_S nanosheets. According to the orthorhombic symmetry of Bi_2_O_2_S (space group: *Pnnm*), the interplanar spacing (*d*) between the (110) and (−110) planes should be identical. However, HRTEM images show that the deviation of the interplanar distances of (110) and (−110) planes is 0.15 Å (Fig. 4c and S10a†). Similarly, the fast Fourier transformation (FFT) image (Fig. S10b†) shows the spots for (110) and (−110) planes and *d*_110_ and *d*_−110_ are non-equivalent by a distance of 0.11 Å. Thus, a visualization of the local atomic distortion is imminent, indicating a breakdown of the local symmetry in Bi_2_O_2_S nanosheets. Similarly, this distortion is also visible for (200) and (020) planes (Fig. 4d). According to the space group, *Pnnm* with crystallographic axes *a* = 3.85 Å; *b* = 3.89 Å and *c* = 11.97 Å, the ratio of *d*_020_ and *d*_200_ ideally should be 1.01 : 1; however the experimentally obtained ratio is 1.075 : 1, which is about 7% higher than the ideal scenario further confirming the local symmetry breakdown in Bi_2_O_2_S nanosheets (Fig. 4d). These spontaneous local distortions change the *D*_2h_ symmetry of the Bi_2_O_2_ chains towards a *C*_2v_ one generating room temperature 2D ferroelectricity in the ultrathin Bi_2_O_2_S nanosheets.

**Fig. 5:**
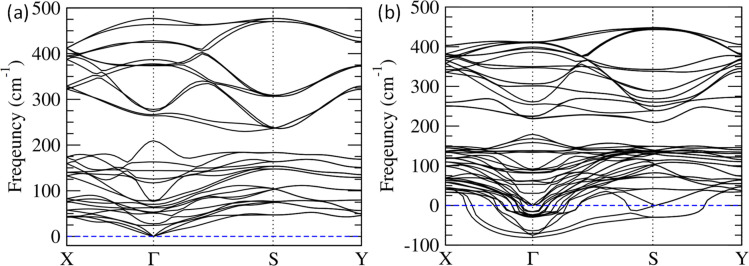
Phonon dispersion of (a) bulk Bi_2_O_2_S and (b) trilayer Bi_2_O_2_S.

To understand the lattice dynamics in Bi_2_O_2_S nanosheets, we calculated phonon dispersion for both bulk and trilayer Bi_2_O_2_S using first-principles DFT calculations (Fig. 5). There is no trace of unstable phonon modes in bulk Bi_2_O_2_S (Fig. 5a). While we move from bulk to trilayer Bi_2_O_2_S, unstable phonon modes are observed throughout the Brillouin zone (Fig. 5b), which indicates local distortion in the nanosheet subsequently causing ferroelectric instability.^54,55^ Interestingly, the negative phonon modes substantially increase at the *Γ* point for the Bi_2_O_2_S monolayer upon the application of stress (Fig. S11, ESI†). This finding corroborates that a minute amount of strain (∼2%) was required to induce ferroelectricity in monolayer Bi_2_O_2_S (Fig. 4a). Furthermore, the unstable phonon modes in trilayer Bi_2_O_2_S are mainly contributed by the Bi and O vibrations as observed from their Eigenvector visualisation (Fig. S12, ESI†). This suggests that the distortion in the (Bi_2_O_2_)^2+^ unit is primarily responsible for breaking the local symmetry in the nanosheets of Bi_2_O_2_S (as visualised in HRTEM) resulting in 2D-ferroelectricity at room temperature.

## Conclusion

In summary, we have successfully employed a simple and easily scalable mild or soft chemistry method to synthesize ultra-thin 2D nanosheets of Bi_2_O_2_S, which can be utilised as a non-toxic and low cost alternative to the charged layered heterostructure, Bi_2_O_2_Se. These nanosheets, obtained through simple solution based synthesis at room temperature, consist of only a few layers, are free-standing, and exhibit a single-crystalline nature. Remarkably, these nanosheets exhibit ferroelectric properties at room temperature, which can be attributed to the slight atomic displacement of the Bi and O atoms in the (Bi_2_O_2_)^2+^ unit, thereby breaking the local inversion symmetry and causing spontaneous dipole moments. Theoretical studies have confirmed that this ferroelectric property is observed in nanosheets with an odd number of layers, as consecutive layers are coupled by an antiferroelectric interaction. This significant finding suggests that the ultra-thin 2D bismuth oxysulphide holds promise for applications in micro/nano-electronic devices in the near future.

## Data availability

All data are available in the manuscript and in the ESI.†

## Author contributions

K. B. conceived the idea and designed the study. R. P., P. D., and K. B. carried out the synthesis, structural, other characterization, and analysis of the data. K. D. carried out the theoretical calculations. A. V., A. G., T. K. M and G. S. carried out AFM and PFM studies. R. S. R. and U. K. G. carried out the TEM study. All authors contributed to writing and editing the manuscript.

## Conflicts of interest

There are no conflicts to declare.

## Supplementary Material

SC-015-D4SC00067F-s001
