# Supplementary material for: Mild chemistry synthesis of ultrathin Bi2O2S nanosheets exhibiting 2D-ferroelectricity at room temperature
Source: Chem Sci. 2024 Apr 9;15(19):7170–7. doi: 10.1039/d4sc00067f (PMC11095514; doi:10.1039/d4sc00067f)
Supplement: SC-015-D4SC00067F-s001 [file SC-015-D4SC00067F-s001.pdf]

Electronic Supplementary Material (ESI) for Chemical Science.
This journal is © The Royal Society of Chemistry 2024

Mild Chemistry Synthesis of Ultrathin Bi_2O_2S nanosheets exhibiting 2D-Ferroelectricity at Room Temperature

Riddhimoy Pathak[a], Prabir Dutta[a], Kapildeb Dolui[b], Aastha Vasdev[c], Adrija Ghosh[a], Raj Sekhar Roy[d], Ujjal K. Gautam[d], Tapas K. Maji[a,e], Goutam Sheet[c], Kanishka Biswas[a, *]

[a] New Chemistry Unit, International Centre for Materials Science and School of Advanced Materials, Jawaharlal Nehru Centre for Advanced Scientific Research (JNCASR), Jakkur P.O., Bangalore 560064, India

[c]Department of Materials Science & Metallurgy, University of Cambridge, 27 Charles Babbage Road, Cambridge CB3 0FS, United Kingdom

[c]Department of Physical Sciences, Indian Institute of Science Education and Research Mohali, Sector 81, S. A. S. Nagar, Manauli, P.O. Box 140306, India

[d] Department of Chemical Sciences, Indian Institute of Science Education and Research Mohali, Sector 81, S. A. S. Nagar, Manauli, P.O. Box 140306, India

[e]Chemistry and Physics of Materials Unit, Jawaharlal Nehru Centre for Advanced Scientific Research (JNCASR), Jakkur P.O., Bangalore 560064, India

**E-mail-ID: kanishka@jncasr.ac.in*

Methods.

Materials. Bismuth nitrate ($Bi(NO_3)_3.5H_2O$, Sigma Aldrich, 99.9%), thiourea ($SC(NH_2)_2$, Sigma Aldrich, 99.9%), potassium hydroxide (KOH, S D Fine-Chem Limited (SDFCL)), sodium hydroxide (NaOH, SDFCL), Disodium EDTA ($C_{10}H_{14}Na_2N_2O_8$, SDFCL) and ethanol were used without any additional purification.

Synthesis. 139 mg (0.2884 mmol) of $Bi(NO_3)_3.5H_2O$, 15 mg (0.206 mmol) of $CS(NH2)_2$ and 428 mg (1.15 mmol) of disodium EDTA were sequentially added at a 10 minutes interval into 30 ml water in a normal laboratory glass beaker. A magnetic bead was placed in the solution and the beaker was placed on a magnetic stirrer to provide continuous uniform stirring to the solution. On the addition of $Bi(NO_3)_3.5H_2O$ into the water, the solution turns into a milky white colour which further turns into an orange-brown colour solution after the addition of $CS(NH_2)_2$. The solution becomes clear after the addition of disodium EDTA. Finally, to the solution, 2.14 gm of KOH is added to make the solution highly alkaline. The solution turns brown on the addition of KOH in the medium. After 20 minutes of stirring, the solution was put to rest which results in the precipitation of the dark brown colour nanosheets. The obtained powders were then washed thoroughly by water and ethanol and vacuum dried at 80° C for 36 hrs. The reaction taking place in the medium occurs in the following way:

Step 1: $Bi(NO_3)_3 + H_2O \rightarrow BiONO_3 + 2H^+ + 2NO_3^-$

In water, $Bi(NO_3)_3$ undergoes hydrolysis to produce $BiONO_3$ and the process of hydrolysis is accelerated in an alkaline medium.

Step 2: $SC(NH_2)_2 + OH^- \rightarrow S^{2-} + H_2NCN + H_2O$

In presence of an alkaline medium, thiourea decomposes into sulphide ions and cyanamide.

Step 3: $2BiONO_3 + S^{2-} \rightarrow Bi_2O_2S + 2NO_3^-$

S^{2-} interacts with $BiONO_3$ to form Bi_2O_2S which is precipitated as a dark brown color product in the aqueous medium.

We have synthesized controlled bulk phase crystal of Bi_2O_2S by sealed tube solid state melting process. Stoichiometric amount of Bi_2O_3 and Bi_2S_3 were taken in a quartz tube and was sealed under high vacuum (10^{-5} Torr). The tubes were slowly heated to 1223 K, annealed for 5 hrs and then slow cooled to room temperature. Shiny crystalline flakes were formed which

was grinded and the indexed PXRD pattern shows pure phase bulk Bi_2O_2S with space group (see Figure S1, SI).

X-ray diffraction (XRD). Powder XRD patterns were collected in Rigaku diffractometer with CuKα radiation (λ = 1.5406 Å) at room temperature. XRD data of the as-synthesized nanosheets were collected after dispersing the nanosheets in ethanol and drop-casting on to a glass slide.

Band gap measurement. Diffuse reflectance spectroscopy was carried out using a Perkin Elmer Lambda 900, UV/VIS spectrometer from which absorption data was calculated using Kubelka-Munk equation: $\alpha/S = (1 - R)^2 /2R$ where α, S and R are the absorption coefficient, scattering coefficient and reflectance, respectively. The optical band gap was then determined from the energy variation of α/S.

X-ray photoelectron spectroscopy (XPS). XPS measurement was carried out using an Omicron nanotechnology spectrometer with an Mg-Kα (1253.6 eV) X-ray source.

Differential Scanning Calorimetry (DSC). A METTLER-TOLEDO differential scanning calorimeter (DSC 822 e) was used to collect DSC data with a ramp rate of 5 K/min in N_2 atmosphere.

Raman Spectroscopy. Room temperature Raman spectra of Bi_2O_2S bulk and nanosheet samples were collected on a Horiba Jobin Yvon LabRAM HR800 spectrometer using a He-Ne laser (632.8 nm).

Dielectric measurements. For dielectric measurements, Bi_2O_2S nanosheets were cold pressed into a pellet. Dielectric properties were then measured in the temperature range from room temperature to 475 K and in the frequency range 1 kHz to 1 MHz with an ac excitation of 300 mV using a Solartron 1296A impedance analyzer.

Transmission electron microscopy (TEM). TEM studies were carried out using a JEOL F200 operated at 200 keV. EDX compositional analysis and elemental mapping were carried in STEM imaging mode. A very dilute solution of Bi_2O_2S nanosheets dispersed in ethanol was drop casted on holey carbon coated Cu grid and used for the TEM studies.

Atomic Force microscopy (AFM) and Piezoresponse force microscopy (PFM). The measurements were done using an Asylum research AFM (MFP–3D) with an additional high voltage amplifier. AFM imaging of the samples were carried out in tapping mode using

AC160TS silicon probes with a nominal tip radius of less than 10 nm. To carry out the PFM measurements, the sample was mounted on a conducting sample holder which was directly connected to the ground of the amplifier. The conductive AFM cantilever having a Pt-Ir tip on it was brought in contact with the sample (nanosheets). An AC excitation of 4V riding on a dc bias voltage (V_{dc}) was applied between the tip and the amplifier ground. The response of the sample to the electrical stimulus was detected through the reflection of the laser beam from the end of the cantilever onto a position-sensitive photodiode. To ensure that the hysteretic effects are due to ferroelectricity which may otherwise arise from electrostatic and electrochemical effects, all the measurements were performed following SS-PFM (switching spectroscopy piezoresponse force microscopy) initiated by Jesse et al.[1,2] In this method, instead of sweeping Vdc continuously, bias is applied in a sequence of pulses where the phase and amplitude measurements are done in the "off" states and an appreciable change is observed in the "off-state" results as compared to the "on-state" measurements which is clear evidence of the minimization of electrostatic effects. We further performed the topographic imaging after the spectroscopic measurements, where no topographic modification was observed which usually occurs due to the electrochemical reaction between the tip and sample.

Density functional theory (DFT) calculations. The first principles electronic structure calculations are performed within the framework of density functional theory (DFT) using generalized gradient approximation (GGA) of the Perdew-Burke-Ernzerhof (PBE)[3] form for the exchange-correlation functional as implemented in the Vinea Ab-initio Simulation Package (VASP).[4] The projector augmented wave (PAW)[5] pseudo-potentials are used to describe the core electrons. Electronic wave-functions are expanded using plane waves up to cut-off energy of 600 eV. Periodic boundary conditions are employed and at least 15 Å slab is used on the surface of few layers to eliminate the interaction between consecutive periodic images. The Monkhorst-Pack k-mesh is set to 11×11 ($11 \times 11 \times 4$) in the Brillouin zone for the self-consistent calculation of few layer cases (bulk), and all atoms are relaxed in each optimization cycle until atomic forces on each atom are smaller than 0.01 eV/Å. As it is well-known that GGA functional underestimates the band gap, all the band structures are computed by using hybrid Heyd-Scuseria-Ernzerhof (HSE06)[6] functional and the GGA-relaxed crystal structure. It should be noted here that we did not include any effect of exciton binding energy in our DFT calculations to estimate the optical band gap. Uniaxial strain is simulated by changing the lattice constant a, whereas the space group P*nnm* is fixed. The symmetric structure represents the equilibrium positions of the atoms at a given strain value. The distorted structure is obtained

by diagonally displacing both the Bi and the S atoms, but in opposite directions from their equilibrium positions. In what follows, if R_{Bi}, and R_S are the equilibrium planer positions of the Bi and S atoms respectively in a symmetric structure, then in the distorted structure, they assume positions at $R_{Bi + \delta}$, and $R_{S - \delta}$, respectively. The outermost S layers are passivated with hydrogen atoms in order to balance the nonstoichiometry due to the additional S layer.

Phonon dispersion of Bi_2O_2S is calculated using density functional perturbation theory (DFPT) as implemented in Quantum Espresso[7]. Here we used the PBE[8] exchange- correlation functional in combination with scalar-relativistic ultrasoft Vanderbilt pseudopotential and energy cutoffs for the plane-waves and change density of 80 and 640 Ry, respectively. We used a q-mesh of 4x4x2 (4x4x1) and a k-mesh of 16x16x4 (16×16×1) for bulk (thin films). The DFT-D3 method[9] is used to include Van der Waals correction.

References

1. Jesse, S.; Baddorf, A. P.; Kalinin, S. V. Switching spectroscopy piezoresponse force microscopy of ferroelectric materials. *Appl. Phys. Lett*. 2006, **88**, 062908.

2. Jesse, S.; Mirman, B.; Kalinin, S. V. Resonance enhancement in piezoresponse force microscopy: Mapping electromechanical activity, contact stiffness, and Q factor. *Appl. Phys. Lett.* 2006, **89**, 022906.

3. Perdew, J. P.; Burke, K.; Ernzerhof, M. Generalized Gradient Approximation Made Simple. *Phys. Rev. Lett*. 1996, **77**, 3865–3868.

4. Kresse, G.; Furthmüller, J. Efficient iterative schemes for ab initio total-energy calculations using a plane-wave basis set. *Phys. Rev. B* 1996, **54** , 11169–11186.

5. Kresse, G.; Joubert, D. From ultrasoft pseudopotentials to the projector augmented-wave method. *Phys. Rev. B* 1999, **59** , 1758–1775.

6. Heyd, J.; Scuseria, G. E.; Ernzerhof, M. Hybrid functionals based on a screened Coulomb potential. *J. Chem. Phys*. 2003, **118**, 8207–8215.

7. Giannozzi, P ; Baseggio, O ; Bonfà, P; Brunato, D; Car, R ; Carnimeo, I; Carlo Cavazzoni, C ; Gironcoli, S; Delugas, P ; Ruffino, F.F ; Ferretti, A; MarzarI, N ; Timrov, I; Urru, A ; Baroni, S. Quantum ESPRESSO toward the exascale. *J. Chem. Phys.,* 2020, **152**, 154105.

8. Perdew, J.P ; Burke, K ; Ernzerhof, M. Generalized Gradient Approximation Made Simple. *Phys. Rev. Lett.,* 1996, **77,** 3865.

9. Grimme, S; Ehrlich, S; Goerigk, L. Effect of the damping function in dispersion corrected density functional theory. *J. Comput. Chem,* 2011, **32,** 1456-1465

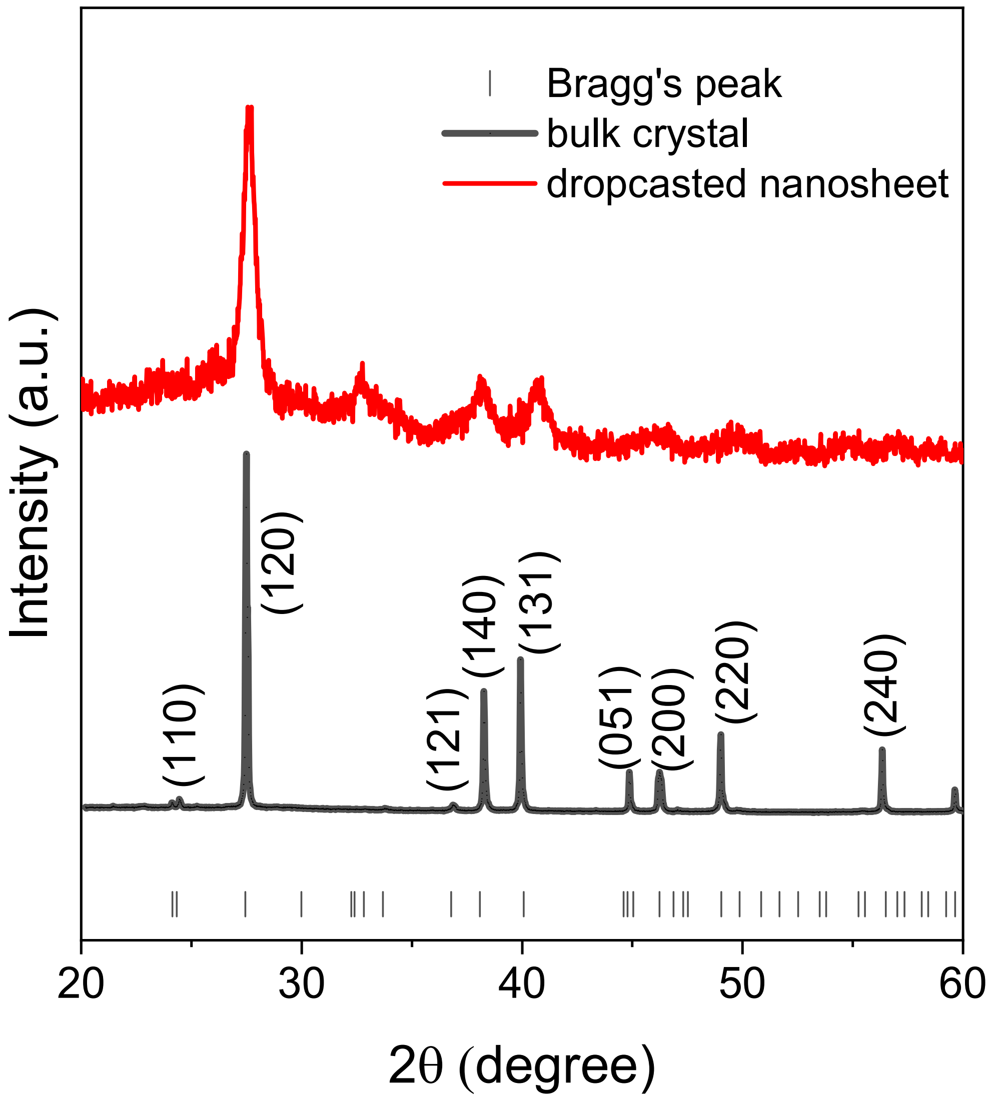

Figure S1. Powder X-ray diffraction patterns of as synthesized bulk crystal of Bi_2O_2S and Bi_2O_2S nanosheets drop casted on a glass slide before vacuum drying.

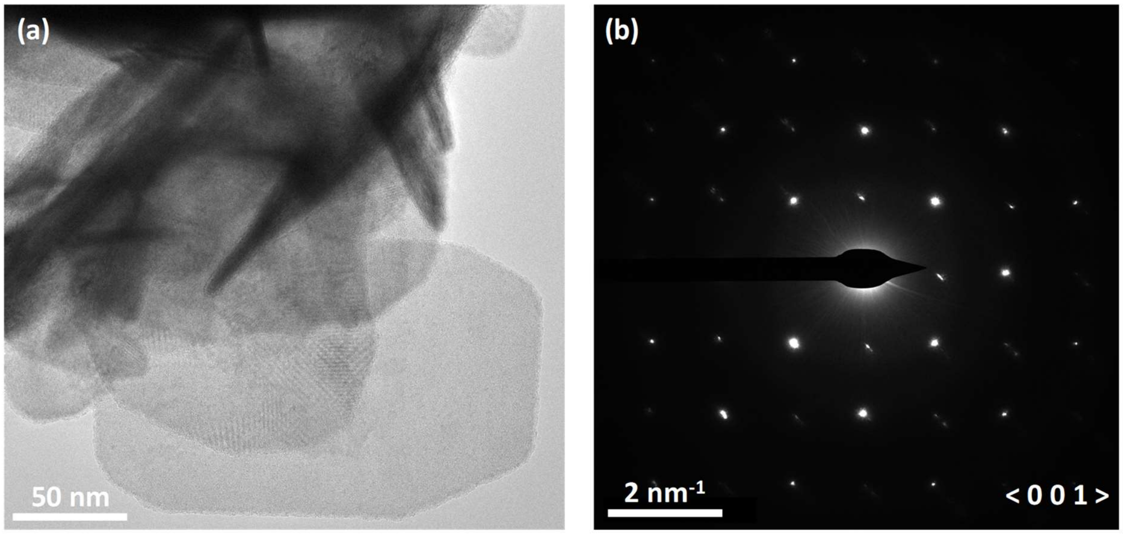

Figure S2. (a) Transmission electron microscopy (TEM) image of Bi_2O_2S nanosheets. (b) Selected area electron diffraction pattern (SAED) of the isolated nanosheet along <001> zone axis

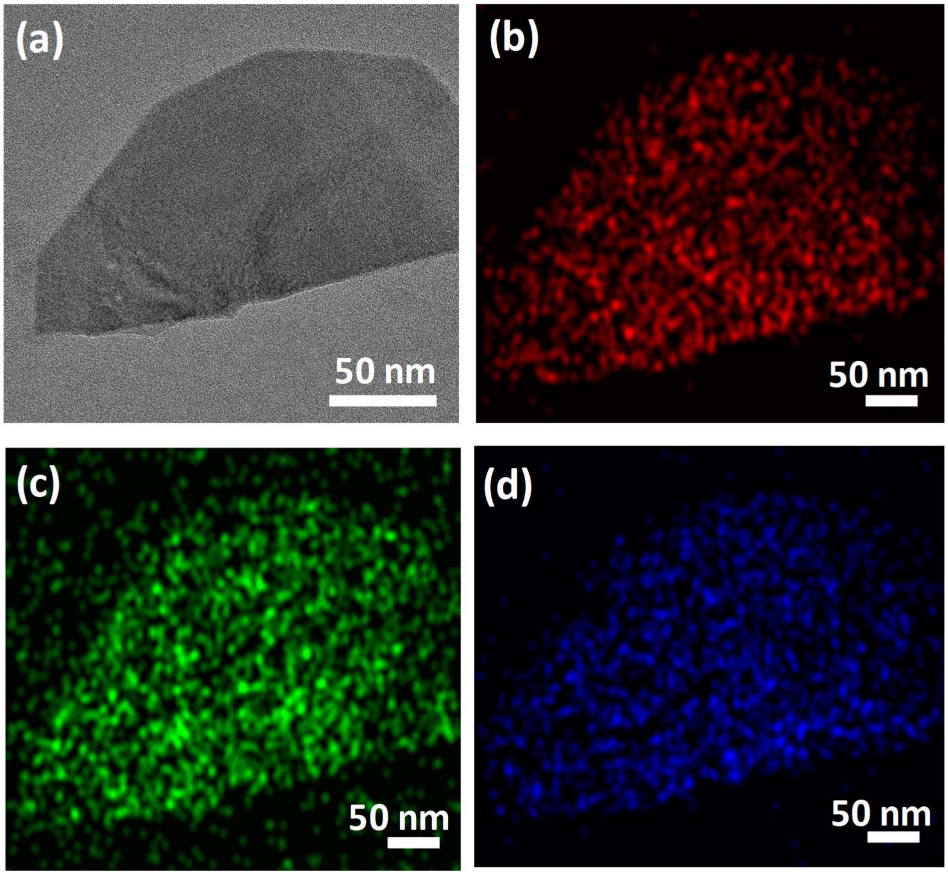

Figure S3. Elemental color mapping of Bi (b), O (c) and S (d) showing homogeneous distribution of the elements in the Bi_2O_2S nanosheet (a).

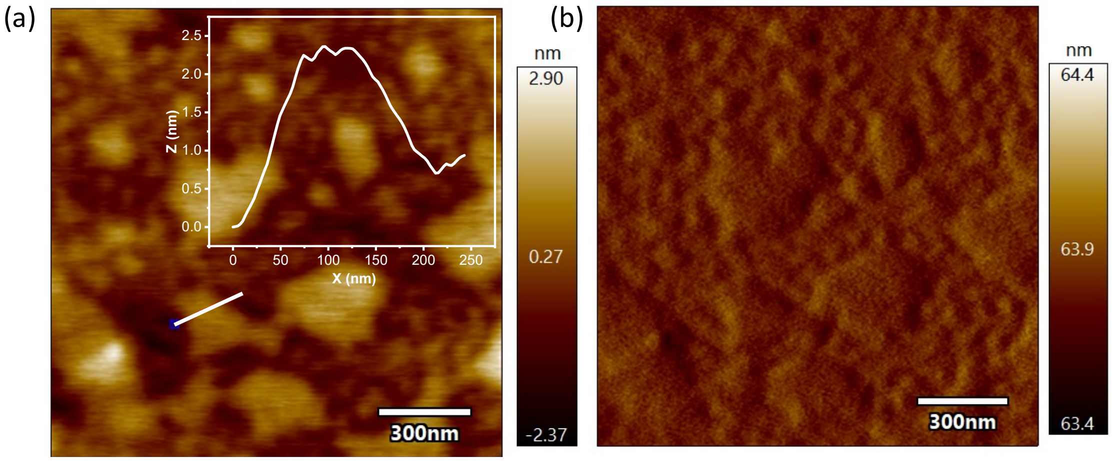

Figure S4. (a) Atomic force microscopy (AFM) and (b) corresponding amplitude image of the thin (~ 2 nm) Bi_2O_2S nanosheet.

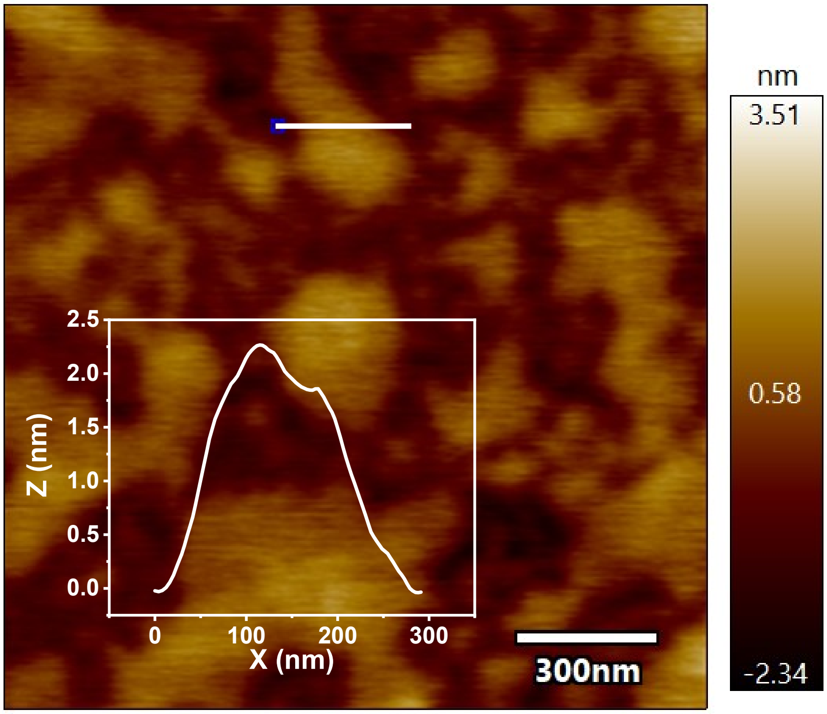

Figure S5: Additional AFM image showing the consistency of thickness (~ 2 nm) of the synthesised Bi_2O_2S nanosheets.

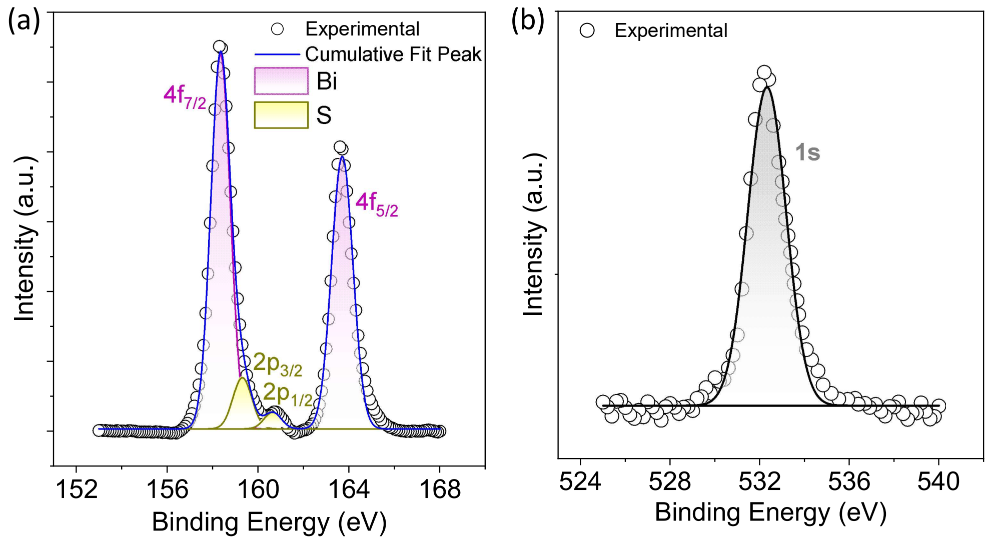

Figure S6. X-ray photoelectron spectra of (a) Bi, S and (c) O.

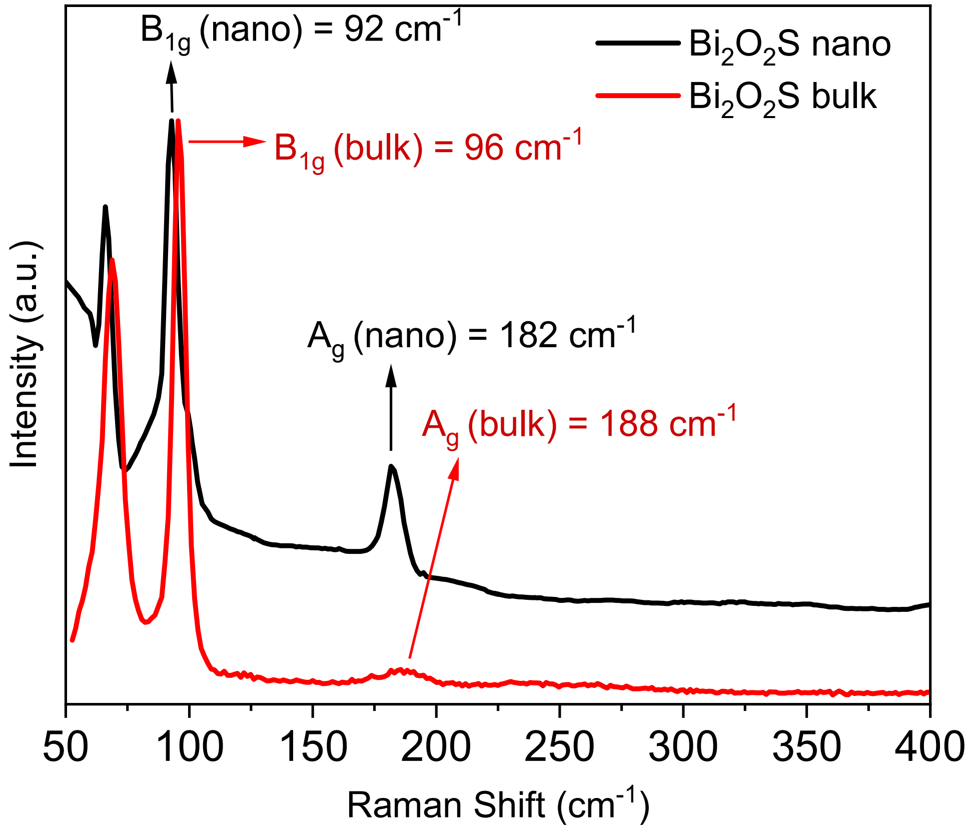

Figure S7. Raman spectroscopy of bulk and nanosheets of Bi_2O_2S.

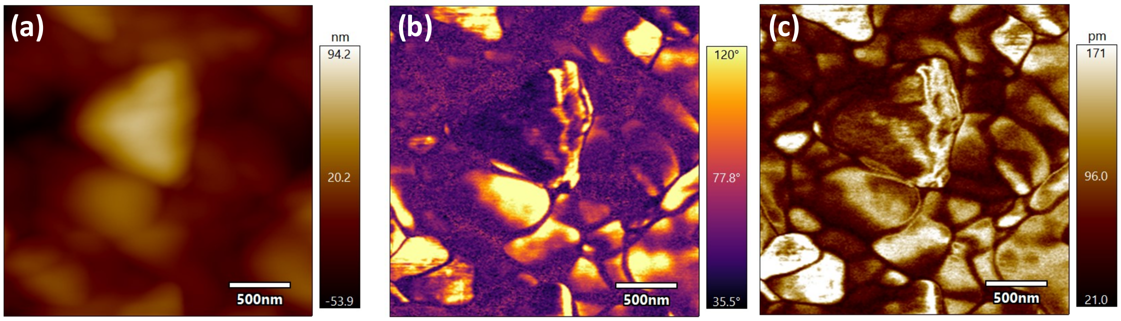

Figure S8. (a) Topography image (b) phase image and (c) amplitude image of Bi_2O_2S nanosheets spin-coated on ITO substrate

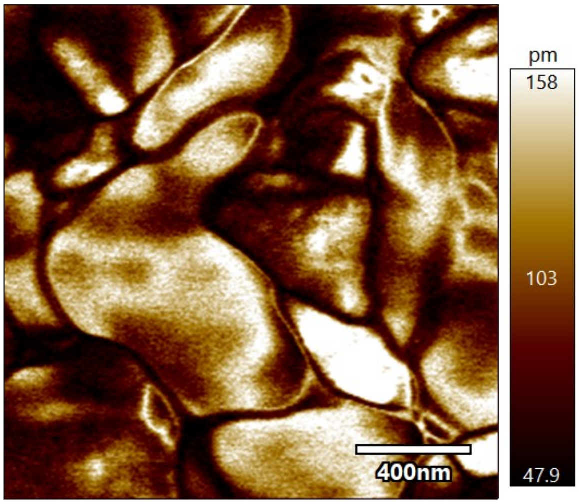

Figure S9. The corresponding amplitude image of Fig 3d of the main manuscript.

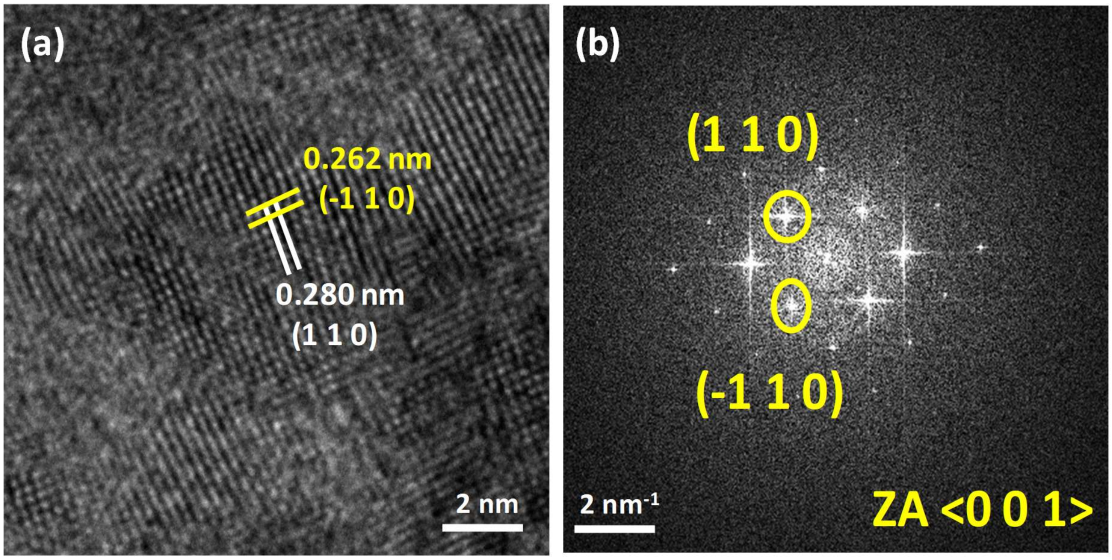

Figure S10. (a) HRTEM image and its corresponding (b) fast Fourier transformation (FFT) image of Bi_2O_2S nanosheet exhibiting deviation between interatomic distances along [110] and [-110] directions.

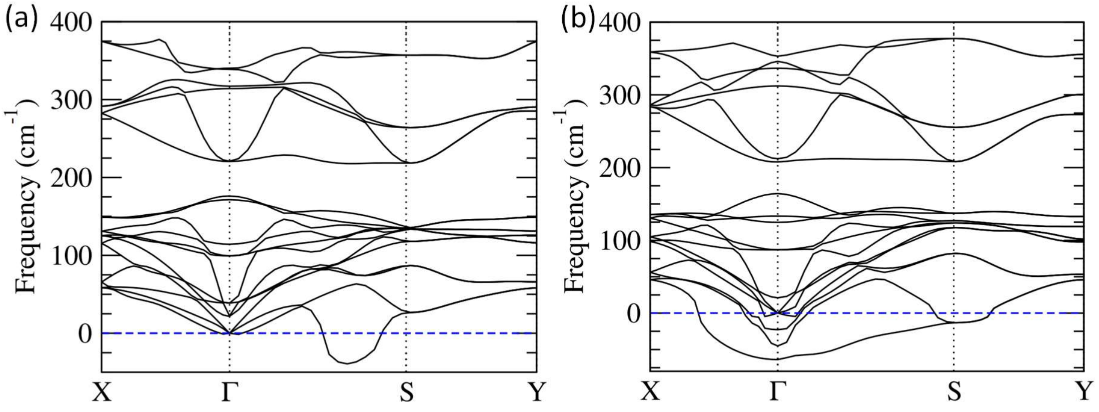

Figure S11. Phonon dispersion of monolayer Bi_2O_2S (a) in absence of any strain and (b) in presence of 2% tensile strain.

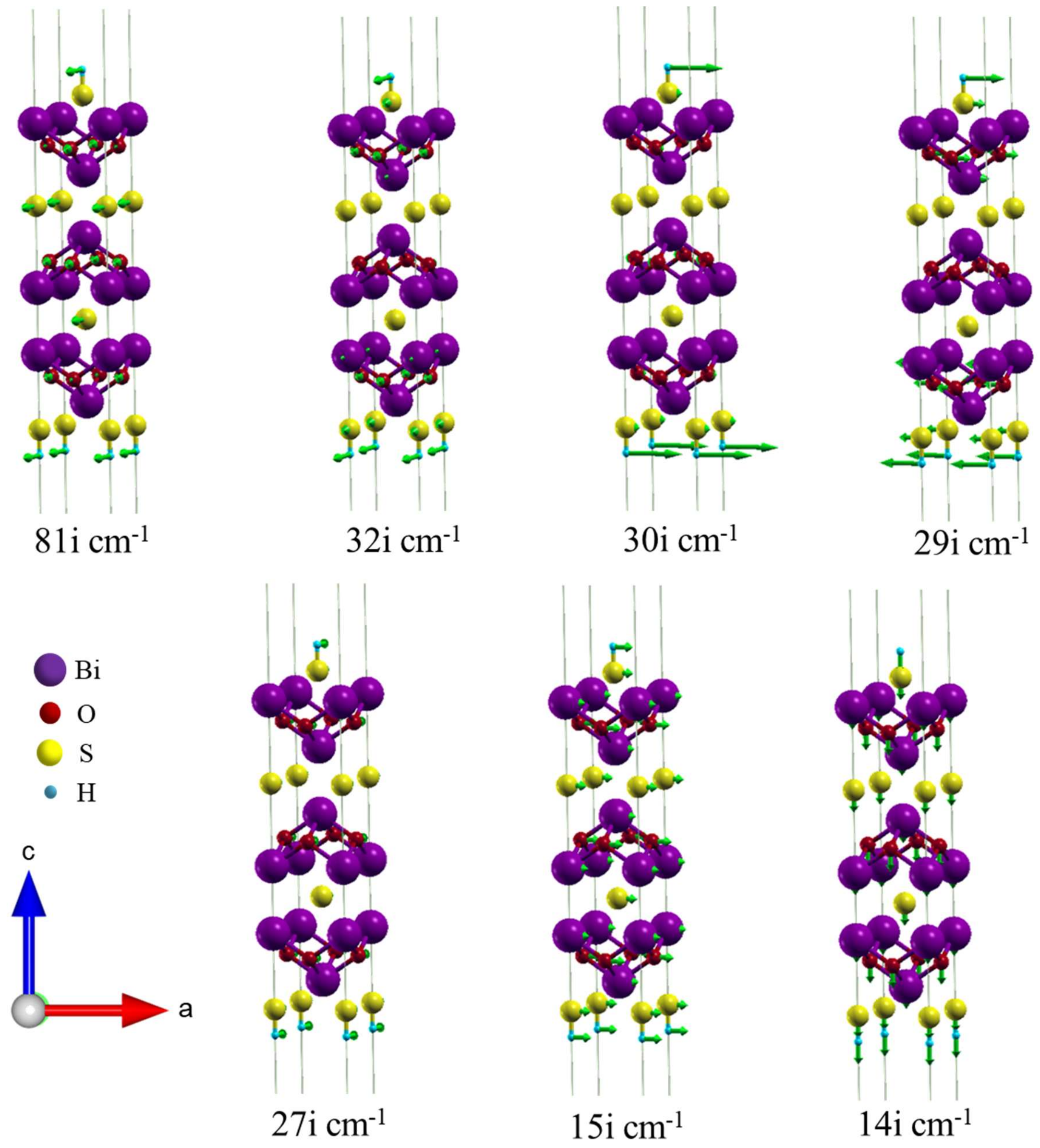

Figure S12. Eigenvector visualisation of the negative modes at Γ point of trilayer Bi_2O_2S involving Bi and O vibrations. (Bi – violet spheres, O – red spheres, S – yellow spheres, H (required for passivation of the charge) – cyan spheres). The green arrows indicate the direction of the vibrations).
